# Expanding Clinician Access to High-Quality, Low-Cost Biomechanical Research: A Technical Report on the Carolina Neurosurgery and Spine Biomechanics Laboratory

**DOI:** 10.7759/cureus.37367

**Published:** 2023-04-10

**Authors:** Ummey Hani, Sam Chewning, Michael Bohl

**Affiliations:** 1 Neurological Surgery, Carolina Neurosurgery and Spine Associates, Charlotte, USA

**Keywords:** biomechanical research, biomechanics, laboratory, simulation models, spine lab

## Abstract

Spine biomechanical research helps us better understand the spine in physiologic and pathologic states and gives us a mechanism by which to evaluate surgical interventions, generate and evaluate models of spine pathologies, and develop novel, data-driven surgical strategies and devices. Access to a biomechanical testing laboratory is therefore potentially invaluable to those who specialize in treating spine pathologies. A number of barriers to access have precluded many clinicians from pursuing their biomechanical research interests, foremost among these is cost. The Carolina Neurosurgery and Spine Biomechanics Research Laboratory (CNSBL) was developed as a model of a low-cost, easy-to-access laboratory capable of generating high-quality data in tests of axial load, tension, torque, displacement, and pathological model testing. Our experience in developing this laboratory suggests that a large number of basic biomechanical research inquiries can be studied in a laboratory composed of less than $7500 USD of hardware. We hope that this model serves as a roadmap for any like-minded practitioners seeking broader access to biomechanical testing facilities.

## Introduction

The last 50 years have seen a dramatic expansion of spine biomechanical testing, underscoring its significance and growing importance in orthopedic and neurosurgical fields [1]. Not only does biomechanical research help us better understand the spine in physiologic and pathologic states, but it also gives us a mechanism by which to evaluate surgical interventions, generate and evaluate models of spine pathologies, and develop novel, data-driven surgical strategies and devices. Access to a biomechanical testing laboratory is therefore potentially invaluable to those who specialize in treating spine pathologies.

Although a large and increasing number of biomechanical research laboratories exist in academic centers and with industry partners around the world, a number of barriers to access have prevented many clinicians from being able to meaningfully pursue their research interests. Foremost is the cost of such laboratories. Notwithstanding the facilities and hardware costs of a laboratory (a six-figure sum for a basic universal testing system), the specimen, tissue handling, and personnel costs of most academic and industry laboratories have significantly limited the types of studies achievable at these centers to those that can acquire extramural funding in the range of $20,000 USD to over $100,000 USD for more complex, larger studies [2]. Studies that do not neatly fit into an industry partner’s financial goals and/or market strategies are unlikely to be supported, and the uncertain process of applying for and managing academic grants can be in and of itself highly limiting to any young physician-scientist looking to initiate a series of investigative efforts in biomechanical research. As such, a model for developing a low-cost, easily accessible biomechanics lab has the potential to broaden biomechanical testing and resource access to a broader community of clinician-scientists and to free those clinician-scientists to pursue their chosen path of investigation regardless of industry or academic grant support. The Carolina Neurosurgery and Spine Biomechanics Research Laboratory (CNSBL) was developed with these goals in mind. This study is a technical report on the development of this laboratory.

## Technical report

Five key elements were identified as essential to the creation of a biomechanical laboratory capable of supporting most basic research efforts: axial load testing, axial tension testing, torque testing, displacement measurement, and a testing medium that is inexpensive and modifiable and has a high biofidelity to human tissue. Testing apparatuses were required to meet the National Institute of Standards and Technology (NIST) certification, provide a platform for a broad range of reproducible scientific analyses, and cost a total upfront investment of less than $7,500 USD. Lab developers furthermore agreed that the laboratory would be available to any members of the larger home institution wanting to pursue an investigative line of biomechanical research regardless of funding status so long as the testing equipment and testing media supported their research goals. Given the relatively low cost compared to academic and industry center labs, it was decided that funding would best be raised privately to preclude financial biases.

Axial load and tension testing

**Figure 1:**
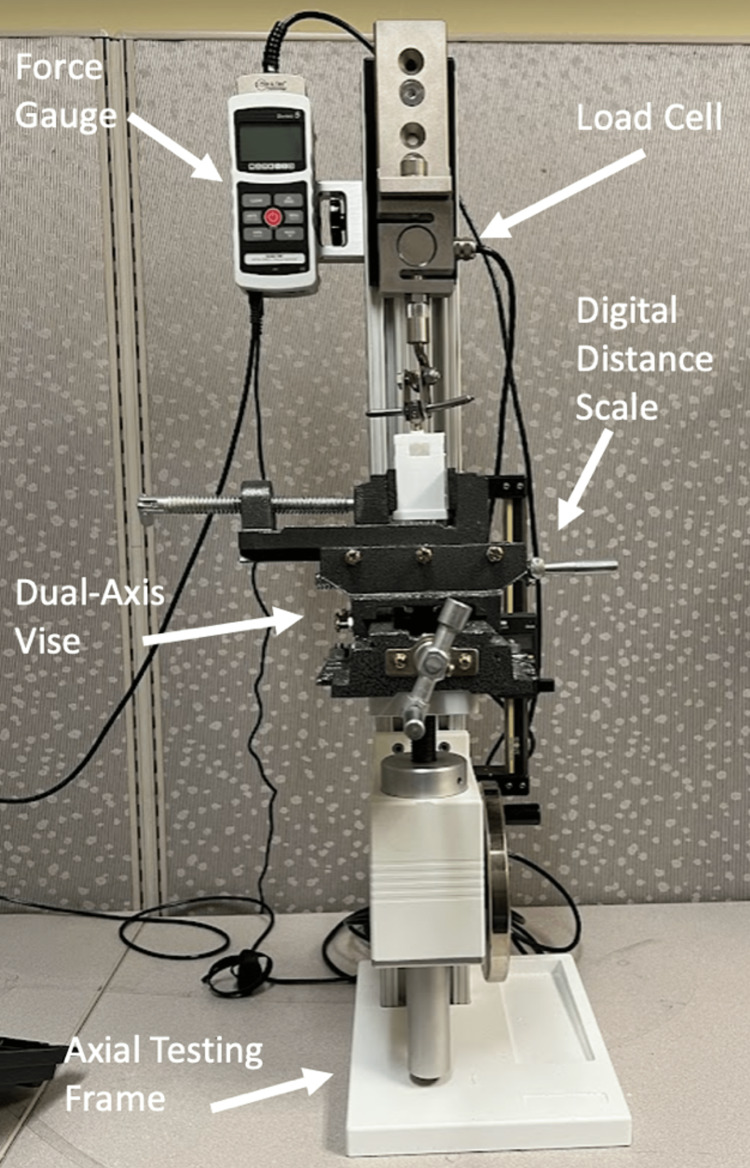
The Mark-10 model Taylor Spatial Frame (TSF) with the Mark-10 series 5 advanced digital force gauge and Mark-10 series R01 tension and compression force sensor* *Mark-10 Corporation, Copiague, New York, USA

A universal testing frame with a force gauge and a sensor is required for axial load and tension testing. Most automated, comprehensive testing solutions for basic axial load and tension testing cost well over $100,000 USD. For this reason, a manual testing frame with NIST certification was selected and customized for our intended use. We chose to purchase a high-capacity manual testing frame that met all of our development criteria with regard to NIST certification, compatibility with a diverse range of testing setups, easy reproducibility, and cost. We found the Mark-10 model TSF frame (Mark-10 Corporation, Copiague, USA) to meet these criteria, with a total cost of $2250 USD (Figure 1) [3]. The same criteria were applied to the selection of a force gauge for measuring and recording force and torque readouts. The Mark-10 series 5 advanced digital force gauge (Mark-10 Corporation, Copiague, New York, USA) is NIST-certified and enables easy transition between axial load and torque sensors. The cost of the force gauge was $1600 USD (Figure 1) [4]. Finally, a load cell calibrated to typical ranges of spine biomechanical testing was required. The Mark-10 series R01 tension and compression force sensor (Mark-10 Corporation, Copiague, New York, USA) calibrated to a maximum of 7500N has NIST certification and is compatible with a wide variety of testing mechanisms. The cost of the force sensor was $635 USD (Figure 1) [5].

Torque testing

**Figure 2:**
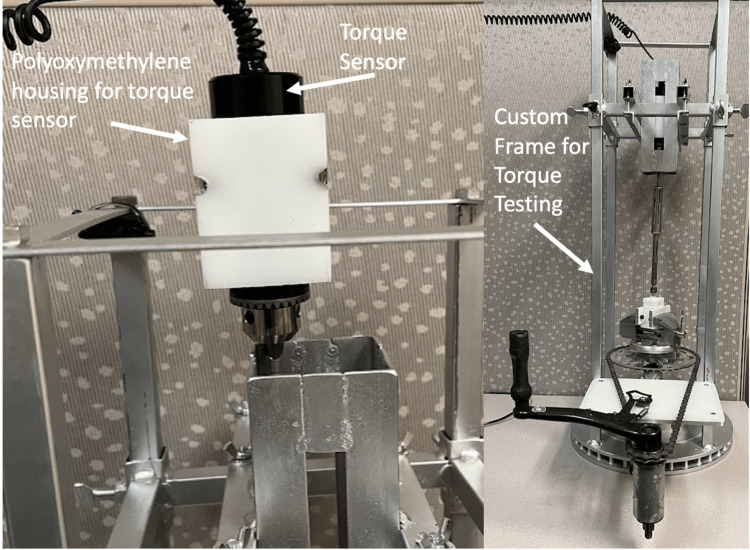
Custom-built testing frame with the Mark-10 series R50 universal torque sensor* *Mark-10 Corporation, Copiague, New York, USA

For the measurement of accurate and reproducible torque measures, we required a NIST-certified force gauge, a bi-directional torque sensor, and a testing frame. The same force gauge used to measure axial load and tension is compatible with torque sensors from the same manufacturer. We were therefore able to use the same force gauge (Mark-10 series 5 advanced digital force gauge) for both axial load testing and torque measurement. The Mark-10 series R50 universal torque sensor calibrated to a maximum of 570 Ncm was selected as our torque sensor, with NIST certification and a cost of $850 USD (Figure 2) [6]. Finally, the universal testing frame used for axial force measurements is not suitable for torque measurement and so the decision was made to devise our own frame (Figure 2). As seen in Figure 2, a frame was devised using spare metal parts from automobiles and bicycles to create a non-rotating component that houses the torque sensor. This component was made of machined polyoxymethylene to custom fit the torque sensor in order to prevent rotation while allowing the torque sensor to freely slide up and down as a test is being completed. The testing media are then secured to a rotating base controlled manually with a hand crank. When measuring the insertional torque of a pedicle screw, the torque sensor and attached pedicle screw will rest atop the testing block. As the base rotates, the pedicle screw is free to move in or out of the testing block while the torque sensor is capturing torque data that are being sent to the digital force gauge. See Figure 2 for a depiction of the novel torque testing frame and its components. Given that the torque testing frame was made of spare metal and plastic parts, we estimated the material cost at less than $100 USD.

Displacement measurement

**Figure 3:**
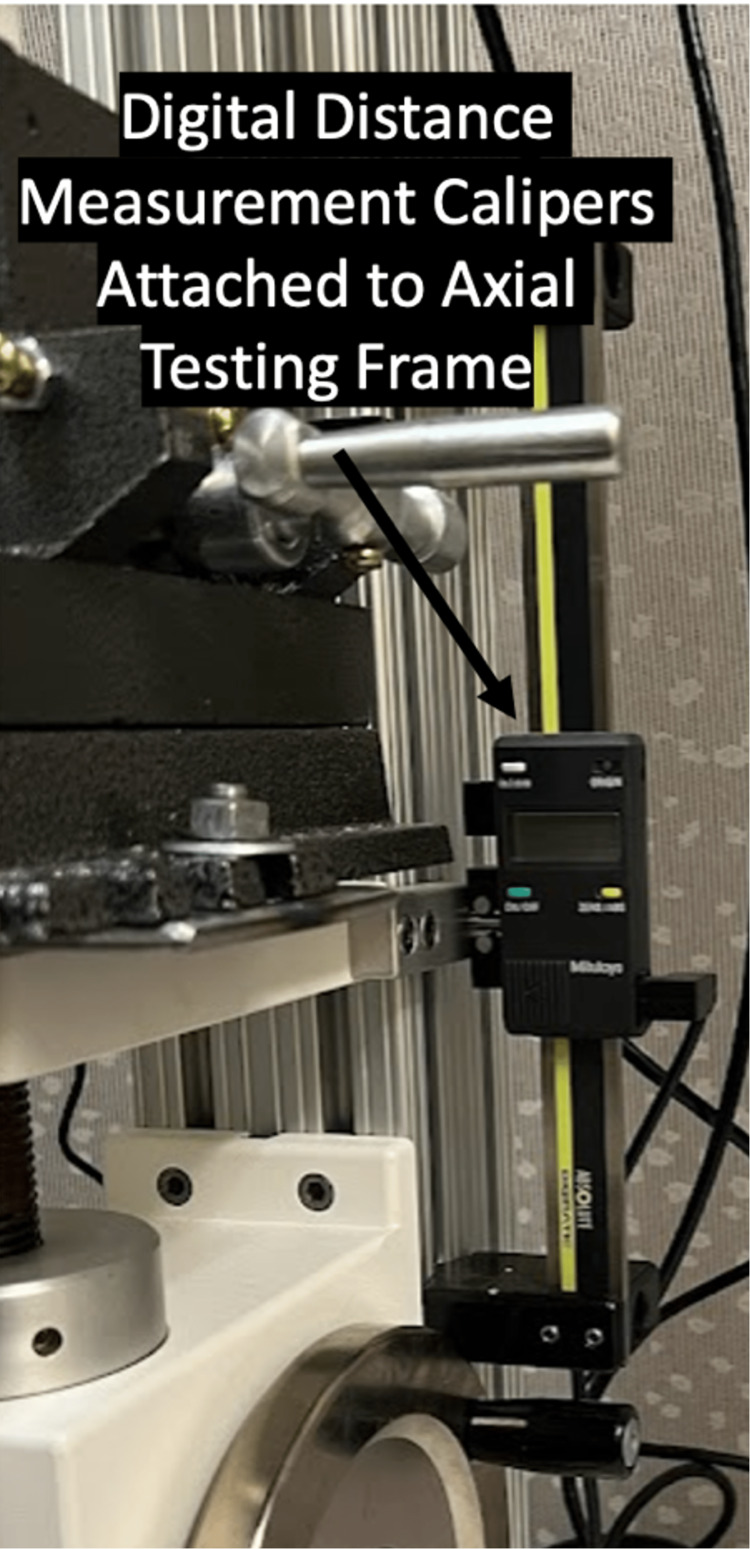
The Mitutoyo vertical digital scale* *Mitutoyo, Kawasaki, Japan

The measurement of certain parameters such as pedicle screw stiffness requires the creation of force over displacement curves. In order to measure force over displacement, we required a digital scale capable of providing continuous distance data output congruous with our axial force data output. For this purpose, we purchased the Mitutoyo vertical digital scale (Mitutoyo, Kawasaki, Japan) (Figure 3) [7]. The scale cost $275 USD, plus additional cables required for data transfer resulting in a total cost of $300 USD. Finally, a software program capable of receiving, storing, and analyzing force, time, and distance data was required. We purchased the Mesur Gauge software package as it was capable of communicating with our force gauge, force sensors, and distance scale. This software package cost $530 USD and functioned appropriately for force and torque over time and force over distance measurements [8].

Testing media

**Figure 4:**
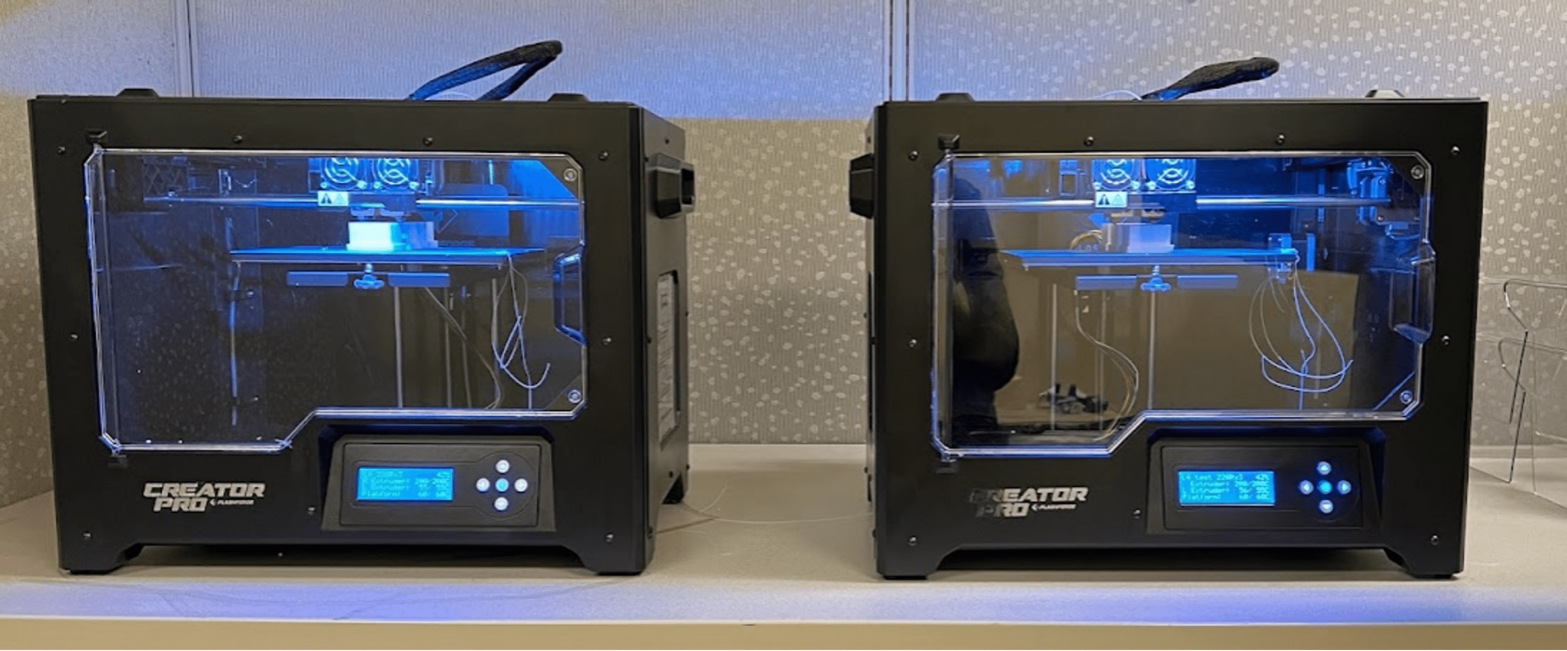
The FlashForge Creator Pro 3D printers* *FlashForge Corp., Zhejiang, China

A growing body of literature has been published on the limitations of cadaveric models for both surgical education and biomechanical research. In addition to their large upfront cost, cadaveric samples require tissue handling and storage facilities, have a limited shelf-life, and are uncontrolled in their anatomy, tissue quality, and presence or absence of confounding pathologies. In short, cadaveric specimens are highly variable and costly. Planning a well-controlled cadaveric study, therefore, requires a larger number of cadaveric specimens in both control and testing groups to account for this variability, adding to the overall cost and confounding variables in cadaveric biomechanical research studies [9,10]. 3D-printed models of human anatomy have been shown to be inexpensive, biofidelic to human bony tissue, and highly controllable and reproducible in terms of anatomy, bone quality, cortico-cancellous architecture, size, haptic feedback, and presence or absence of any desired pathologies [11-18]. As such, we chose 3D-printed bone models to be our primary medium for biomechanical testing. 3D printers and filaments are required to create 3D-printed models of human anatomy. We chose a 3D printer that has been described in the literature as being suitable for creating models for biomechanical research, namely, the FlashForge Creator Pro (FlashForge Corp., Zhejiang, China) [17]. These printers currently cost $300 USD. The software required to run these printers is free and available online, as are software programs that enable the creation and modification of computerized 3D models for use in testing [19]. Given their low cost, we decided to purchase two 3D printers to enable faster creation of testing media to eliminate this potential roadblock to more efficient study completion (Figure 4). Given the hardware costs described above, the total cost of our biomechanical research laboratory was $6865 USD (Table 1).

**Table 1:** Hardware costs of the Carolina Neurosurgery and Spine Biomechanical Research Laboratory (CNSBL)

Hardware	Cost
Manual testing frame	$2250
Force gauge	$1600
Force sensor	$635
Torque sensor	$850
Torque testing frame	$100
Digital distance scale and cables	$300
Software	$530
3D printers x2	$600
TOTAL	$6865

## Discussion

**Figure 5:**
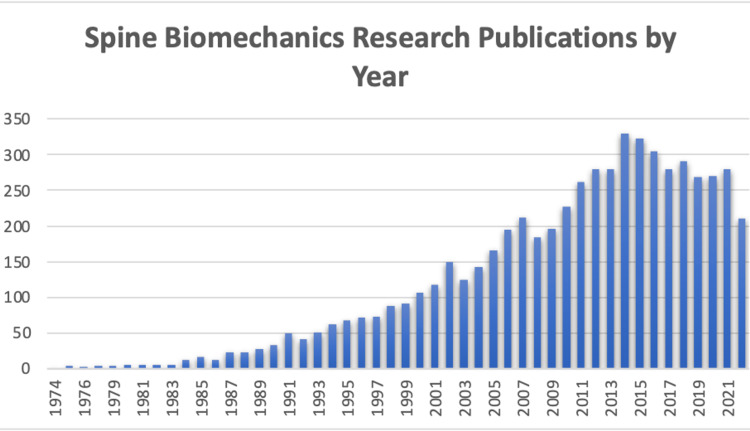
Spine biomechanics research publications by year

Clinicians treating spine pathologies are best suited for identifying unmet needs and gaps in our knowledge about spinal diseases and their treatment. Identification of our field’s unmet needs can potentially lead to the advancement of our field if clinicians are encouraged and enabled to generate lines of data-driven research from their clinically based observations. While the last five decades have seen a dramatic expansion in spine biomechanical testing (Figure 5), the majority of clinicians seeing patients with spinal pathologies do not have access to a biomechanical research laboratory, and those that do are often stymied by the overbearing logistics of acquiring extramural funding to support a cadaveric study in a multi-million-dollar research laboratory [1]. Creating a model for developing low-cost, high-quality biomechanical research laboratories has the potential to transform our field by expanding biomechanical research access to a much broader group of clinicians and to more readily incorporate these resources into the day-to-day clinical practices of neuro and orthopedic surgeons and their associated colleagues. The ability to quickly translate in vivo clinical observations to in vitro laboratory analysis and testing affords a tremendous opportunity to develop new bedside techniques, procedures, or devices in the diagnosis and treatment of spine disease. The above-reported model provides a roadmap for the development of such a laboratory, all at a cost of less than $7500 USD.

One potential limitation to the expansion of the above research laboratory model is that our development team consisted of team members with significant engineering and machining skill sets, enabling the creation of homemade, less-expensive testing equipment. This specifically refers to our development of a testing frame for torque testing. For an additional $2780 USD, an industry-manufactured torque testing frame can be purchased, leaving a total upfront laboratory cost of less than $10,000 USD. This still represents a sum much more easily achieved which lends to broader access and utility than existing academic or industry-based labs. Nevertheless, to make the most of any biomechanical research laboratory, some amount of engineering and manufacturing ingenuity will be required. Funding to support a full- or part-time laboratory engineer is absolutely an advantage, but for most research inquiries, this cost can be avoided if the clinicians leading the investigation are willing to learn and innovate basic engineering solutions to their study methodologies.

Future directions for this work include a validation study to demonstrate the reliability of our study apparatuses, for which data collection is already underway. Work has also begun on a novel new method for reducing variability in biomechanical research studies, with promising preliminary results. Additional planned studies include the development of novel models of various spine pathologies, as well as a series of novel cranial biomechanical investigations. The ability of low-cost laboratories like the CNSBL to expand testing beyond spine pathologies to other regions of neurosurgical interest such as cranial fixation techniques and systems is largely unexplored, and we are eager to provide this opportunity to our cranial neurosurgery colleagues. These are a few of the many future investigatory efforts we intend to pursue in our low-cost, easy-access biomechanical laboratory, and we hope that our experience inspires other like-minded clinicians to pursue the development of the same resources.

## Conclusions

Many clinicians treating spine pathologies are precluded from pursuing their clinically derived lines of biomechanical research due to the existing cost and accessibility challenges posed by academic and industry-based biomechanical research laboratories. In this technical report, we describe a model for building a low-cost, high-quality biomechanical research laboratory intended to expand access to these resources to a much wider population of clinicians. By expanding access to biomechanical testing resources, we hope to advance the fields of surgical and conservative spine care by enabling those clinicians with the closest access to patients to more easily convert their clinical observations to lines of biomechanical research testing. Our experience in developing this laboratory suggests that a large number of basic biomechanical research inquiries can be studied in a laboratory composed of less than $7500 USD of hardware. We hope that this model serves as an inspiration and potentially a roadmap for any like-minded practitioners seeking broader access to biomechanical testing facilities.
