# Correction: Expanding Clinician Access to High-Quality, Low-Cost Biomechanical Research: A Technical Report on the Carolina Neurosurgery and Spine Biomechanics Laboratory

**DOI:** 10.7759/cureus.c298

**Published:** 2025-09-16

**Authors:** Ummey Hani, Sam Chewning, Michael Bohl

**Affiliations:** 1 Neurological Surgery, Carolina Neurosurgery and Spine Associates, Charlotte, USA

The Ethics Statement and Conflict of Interest Disclosures section has been updated to disclose the following:

**Financial relationships:** All authors declare(s) employment from Carolina Neurosurgery and Spine Associates.

